# LSD-induced increases in social adaptation to opinions similar to one’s own are associated with stimulation of serotonin receptors

**DOI:** 10.1038/s41598-020-68899-y

**Published:** 2020-07-22

**Authors:** Patricia Duerler, Leonhard Schilbach, Philipp Stämpfli, Franz X. Vollenweider, Katrin H. Preller

**Affiliations:** 1 Neuropsychopharmacology and Brain Imaging, Department of Psychiatry, Psychotherapy and Psychosomatics, University Hospital for Psychiatry Zurich, Lenggstrasse 31, 8032 Zürich, Switzerland; 2 Independent Max Planck Research Group for Social Neuroscience, Max Planck Institute of Psychiatry, Kraepelinstr. 2-10, 80804 Munich, Germany; 3 Department of Psychiatry, Ludwig-Maximilians-Universität, Munich, Germany; 4 Clinic for Disorders of Social Interaction, LVR Klinikum Düsseldorf/Kliniken der Heinrich-Heine-Universität Düsseldorf, Bergische Landstr. 2, 40629 Düsseldorf, Germany; 5 Department of Psychiatry, Psychotherapy and Psychosomatics, University Hospital for Psychiatry Zurich, Lenggstr. 31, 8032 Zurich, Switzerland

**Keywords:** Chemical biology, Neuroscience, Psychology

## Abstract

Adapting one’s attitudes and behaviors to group norms is essential for successful social interaction and, thus, participation in society. Yet, despite its importance for societal and individual functioning, the underlying neuropharmacology is poorly understood. We therefore investigated its neurochemical and neural correlates in a pharmacological functional magnetic resonance imaging study. Lysergic acid diethylamide (LSD) has been shown to alter social processing and therefore provides the unique opportunity to investigate the role of the 5-HT_2A_ receptor in social influence processing. Twenty-four healthy human volunteers received either (1) placebo + placebo, (2) placebo + LSD (100 µg), or (3) the 5-HT_2A_ receptor antagonist ketanserin (40 mg) + LSD (100 µg) at three different occasions in a double-blind, randomized, counterbalanced, cross-over design. LSD increases social adaptation but only if the opinions of others are similar to the individual’s own. These increases were associated with increased activity in the medial prefrontal cortex while participants received social feedback. Furthermore, pretreatment with the 5-HT_2A_ antagonist ketanserin fully blocked LSD-induced changes during feedback processing, indicating a key role of the 5-HT_2A_ system in social feedback processing. Our results highlight the crucial role of the 5-HT-system in social influence and, thus, provide important insight into the neuropharmacological basis of social cognition and behavior.

## Introduction

The quality and quantity of individuals' social relationships has been tightly linked to morbidity and mortality^1^. Social influence is omnipresent in our everyday life and reacting to the opinions of others is deeply rooted in human nature^2^. Every time people interact or communicate, be it in real life or virtually, people are influenced by each other. Social adaptation refers to the act of changing one’s attitudes, beliefs, or behaviors to match social norms that are implicitly or explicitly shared by a group of individuals^3^. Zaki et al.^4^ suggest that exposure to social norms directly influences participants’ neural representations of the value they assign to stimuli. Being susceptible to social influence is essential for successful social interaction and communication, and also for the functioning and organization of an entire society. However, it can also have negative consequences if misused^5,6^.

In recent years, social neuroscience has advanced our understanding of the neural systems underlying social influence processing^7–9^. Amongst other brain regions, it has been shown that frontal regions as well as the reward system are strongly implicated in adaptation to group norms^10–12^. In particular, when exposed to a mismatch between the own and the group opinion, participants showed a deactivation of the ventral striatum and an activation of medial frontal areas^8,10,13^, brain regions associated with conflict detection, reinforcement learning, and social cognition^2,8,10,11,14,15^.

So far, most studies have focused on localizing brain regions implicated in social influence processing. However, neuropharmacological studies investigating processes of social adaptation are rare. While dopamine and oxytocin have been shown to influence certain aspects of social adaptation^16–18^, the role of the serotonin (5-HT) system in humans in these processes is currently unknown. Yet, the 5-HT system has repeatedly been shown to modulate social cognition and is implicated in various psychiatric disorders and their treatment^19–22^. This is particularly important given that therapeutic approaches in psychiatry always involve social elements^23^ and that psychiatric disorders have recently been described as disorders of social interaction^24,25^.

The classic psychedelic lysergic acid diethylamide (LSD) has predominantly agonistic activity at 5-HT2A/C, -1A/B, -6 and -7 and dopamine (D) D1 and D2 receptors^26–28^. Pretreatment with the selective 5-HT_2A_ antagonist ketanserin has been shown to block its subjective effects^29^. Therefore, LSD, particularly in combination with ketanserin, offers the unique opportunity to investigate the role of the 5-HT_2A_ receptor system in cognitive processes. Importantly, psychedelic substances are known to modulate social behavior and interaction, as well as brain activity and connectivity in areas related to social processing such as the medial prefrontal cortex (mPFC)^29–31^. A recent preliminary study in ten participants reported increased suggestibility on a cued mental imagery task after LSD administration^32^. In the early 1950′s LSD’s potential suggestibility-enhancing effect was also the focus of the Central Intelligence Agency (CIA)’s MK Ultra project, testing LSD for ‘mind control’ and chemically assisted interrogation. Despite these unethical experiments, the efficacy of LSD for the purpose of ‘mind control’ has never been shown^33^ and controlled experiments regarding LSD’s effect on social influence processing and their underlying pharmacology are still lacking.

In this study we therefore investigated the impact of Placebo (Pla), LSD, and LSD after pre-treatment with ketanserin on social influence processing and social decision-making and its underlying neuropharmacology in an aesthetic judgment task. While undergoing functional magnetic resonance imaging (fMRI), participants judged the aesthetic quality of art pictures. After that, they were confronted with a group norm, and subsequently re-evaluated their decision. This newly developed task has various advantages over previously employed paradigms: (1) it does not rely on expert knowledge, but is based on opinions, (2) it is assessing both, social norm feedback processing and subsequent social decision-making in the MRI scanner, and therefore offers the opportunity to disentangle these processes, (3) it allows to vary the magnitude of disagreement between participants’ opinions and norm group judgements.

Based on previous findings^32^ we hypothesized that LSD increases social adaptation compared to Pla. We furthermore hypothesized that LSD affects both behavioral adaptation as well as activity in brain areas relevant for social influence processing^4^. Additionally, we hypothesized that LSD-induced changes in social influence processing would be at least partially blocked by ketanserin. Lastly, given the relationship between personality and social cognition^34^, we hypothesized that the magnitude of LSD-induced effects on social adaptation depends on baseline personality and sociability.

## Results

### Subjective effects

Detailed results of subjective drug effects assessed with the Altered States of Consciousness (5D-ASC) questionnaire and the Positive and Negative Affect Schedule (PANAS) questionnaire are reported in the supplementary material (Supplementary Results, Figs. S1 and S2). In summary, all LSD-induced subjective drug effects were blocked by ketanserin.

### Social influence paradigm

#### Rating 1 (R1)

A repeated measures ANOVA for the initial rating of the pictures revealed a significant main effect for treatment (F(2, 142) = 6.12, p < 0.01). Bonferroni corrected simple main effect analyses revealed a significant difference between LSD and Pla treatment conditions and the LSD and Ket + LSD conditions for R1 (all p < 0.05). The R1 scores did not differ significantly between the Pla and the Ket + LSD treatment conditions (p > 0.05) (Fig. S3).

#### Weight of advice (WOA) score

**Figure 1:**
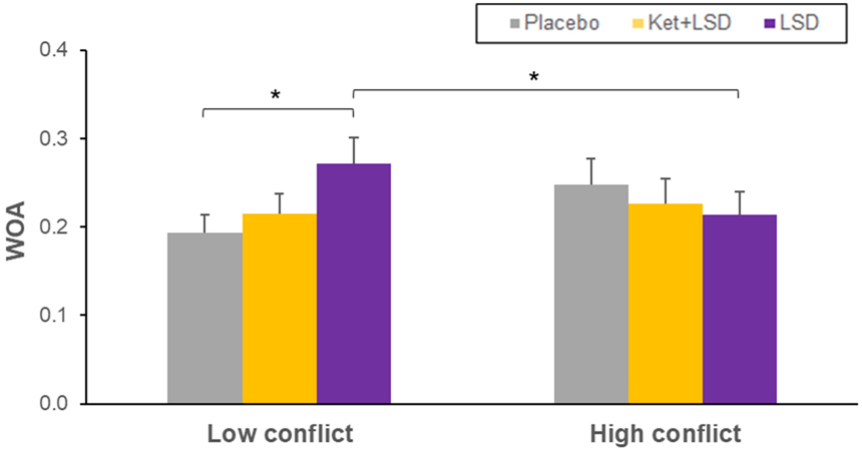
Weight of advice (WOA). The WOA scores represents the adaptation to the group norm in the Pla, Ket + LSD and LSD treatment conditions. A score close to 1 reflects a strong adaptation to the group norm. Participants showed the strongest adaptation to the group norm in the LSD LC condition which was significantly different from the Pla LC and from the LSD HC conditions. *p < 0.05, Sidak-corrected *n* = 24.

The WOA Score represents the adaptation to the group norm^13^. A repeated measures ANOVA (treatment × condition) for the WOA score revealed a significant interaction for treatment × condition (F(2, 46) = 4.47, p < 0.05). Sidak-corrected simple main effect analyses showed a significant difference between WOA scores in the LSD Low Conflict (LC) and the Pla LC conditions (p = 0.01) indicating more adaptation to the group norm in the LSD LC compared to the Pla LC condition. Furthermore, there was a significant difference within the LSD treatment condition between the LC and High Conflict (HC) condition (p = 0.01) with more adaptation to the group norm in the LC condition. Within the Pla condition, there was no significant difference between the LC and HC conditions (p = 0.1). There were no further significant differences within or between treatment conditions after Sidak-correction (all p > 0.05; Fig. 1). Means, standard deviations, and effect sizes are listed in the supplementary material (see Table S1).

#### fMRI data

##### Placebo condition

**Figure 2:**
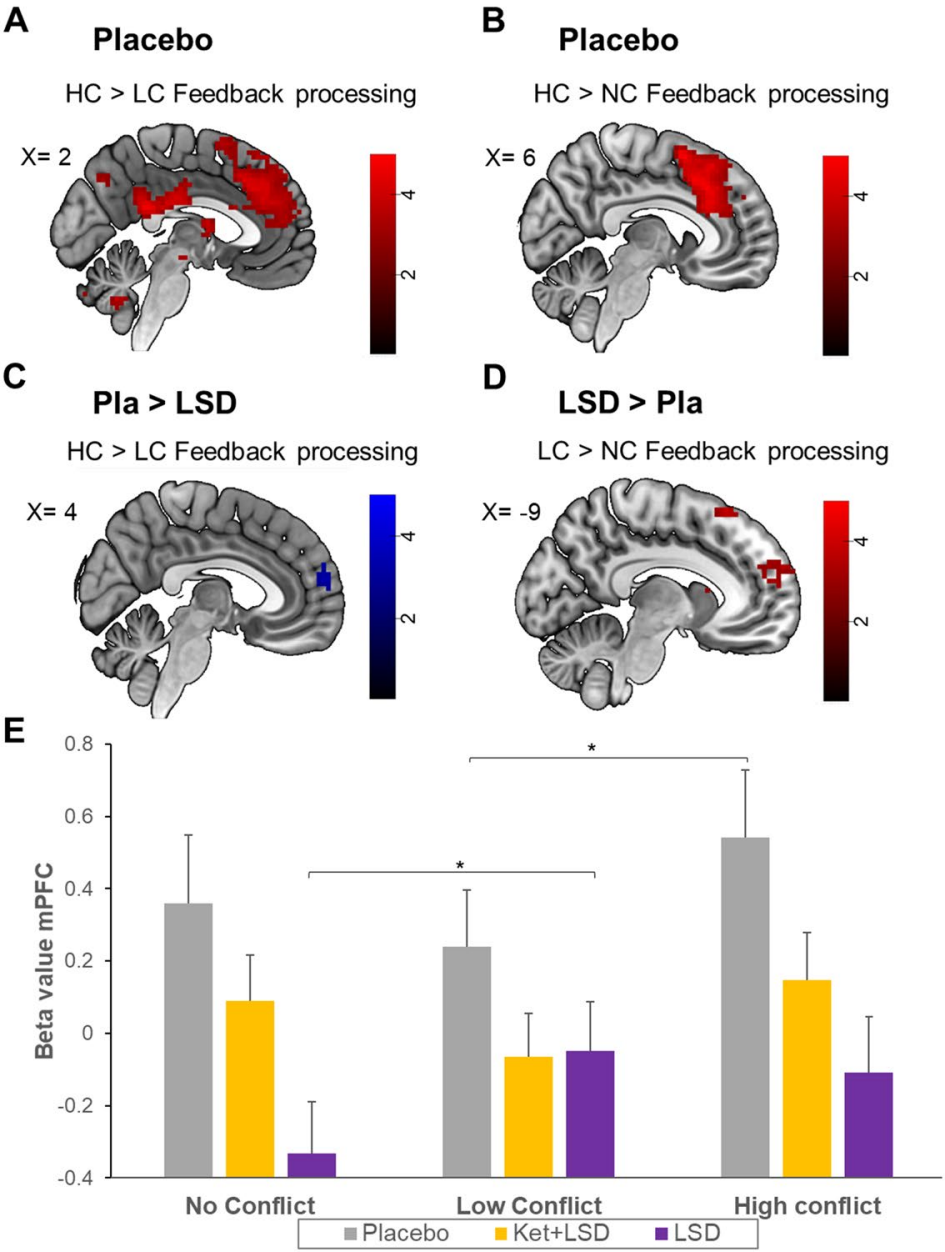
Feedback processing in the Placebo and in the LSD compared to the Placebo condition. Differences in BOLD signals for (**A**) HC > LC contrast for the feedback processing (FB) for Pla at peak in the mPFC voxel (x = − 3, y = 47, z = 25); (**B**) HC > NC FB contrast for Pla at peak SMA voxel (x = 6, y = 29, z = 52); (**C**) HC > LC FB contrast for Pla > LSD at peak mPFC voxel (peak: x = 0, y = 59, z = 19); (**D**) LC > NC FB contrast for LSD > Pla at peak mPFC voxel (peak: x = − 9, y = 62, z = 25); p < 0.05 FWE corrected (peak level after SVC). Red Shades represent an increase in BOLD signal. Blue shades represent an LSD-induced decrease in BOLD signal. Colorbars indicate t-values. Data displayed at p < 0.005, k > 10 (uncorrected). *n* = 24. (**E**) Comparison of Beta values in the mPFC for all conditions and treatments. Data are displayed as mean and standard error of the mean. *p < 0.05, Bonferroni corrected.

For the HC > LC contrast, we found a significant effect during feedback (FB) processing of the group rating in the mPFC (x = − 3, y = 47, z = 25, k = 70, T = 3.74), the supplementary motor area (SMA) (x = 9, y = 20, z = 49, k = 44, T = 4.61), the right nucleus accumbens (x = 30, y = 23, z = − 5, k = 15, T = 4.12) and the precuneus (x = 0, y = − 70, z = 40, k = 10, T = 3.23; Fig. 2A). The HC > No Conflict (NC) contrast revealed a greater BOLD signal during FB processing in the SMA (x = 6, y = 29, z = 52, k = 36, T = 4.54; Fig. 2B). HC > NC during rating 2 (R2) revealed a significantly higher BOLD signal in the angular gyrus (x = 42, y = − 79, z = 28, k = 10, T = 3.49; see Fig. S4A) (all p < 0.05 FWE corrected after SVC; Table S2).

##### Placebo vs. LSD condition

During FB processing, the contrast HC > LC revealed a greater BOLD signal in the mPFC (x = 0, y = 59, z = 19, k = 32, T = 3.93) in the Pla condition compared to LSD (Fig. 2C). However, significantly increased BOLD signal in the mPFC (x = − 9, y = 62, z = 25, k = 34, T = 3.12) was found in LSD condition for the LC > NC contrast during feedback processing (p < 0.05 FWE corrected after SVC; Fig. 2D). No differences were found during subsequent decision-making (R2).

To further investigate which changes underlie the interaction effects described above, beta values were extracted from the mPFC ROI for all conditions. A repeated measures ANOVA revealed a significant main effect for drug (F(2, 94) = 5.02, p < 0.01) and condition (F(2, 94) = 3.89, p < 0.05) and a significant interaction for drug × condition (F(4, 188) = p < 0.005). Simple main effect analyses comparing within-treatment condition effects revealed a significant increase from LC to HC in the Pla condition (p < 0.001), and a significant increase from NC to LC in the LSD condition (p < 0.001). No further within-treatment comparisons reached significance (all p > 0.05, Fig. 2E).

##### Ket + LSD vs. LSD condition

**Figure 3:**
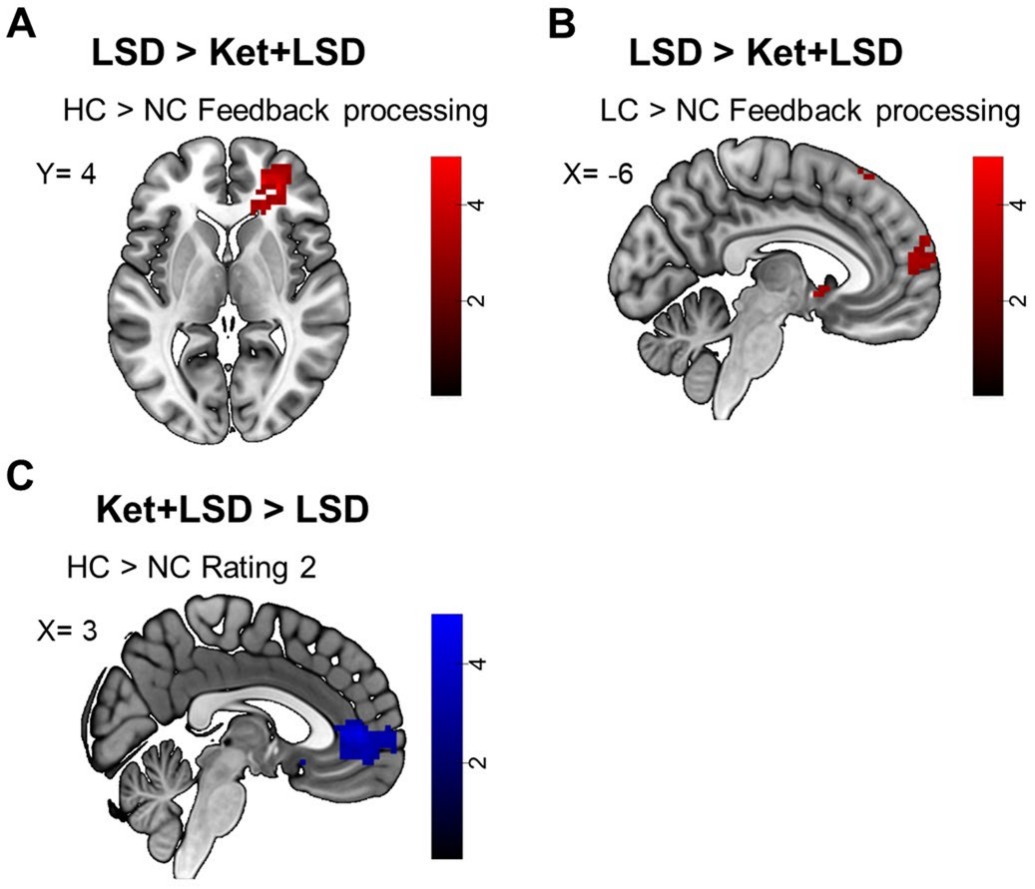
fMRI data. Differences in BOLD signal for (**A**) HC > NC FB contrast for LSD > Ket + LSD at peak inferior frontal gyrus voxel (peak: x = 30, y = 44, z = 4); (**B**) LC > NC FB for LSD > Ket + LSD at inferior frontal gyrus voxel (peak: x = 30, y = 41, z = 4); (**C**) HC > NC Rating 2 contrast for Ket + LSD > LSD at peak medial OFC voxel (peak: x = 3, y = 47, z = 4); p < 0.05 FWE corrected (peak level after SVC); Blue shades represent an LSD-induced decrease in BOLD signal. Red Shades represent an LSD-induced increase in BOLD signal. Colorbars indicate t-values. Data displayed at p < 0.005, k > 10 (uncorrected). *n* = 24.

Comparing the HC > NC contrast during FB processing between LSD and Ket + LSD treatment conditions revealed a significant higher BOLD signal in the lateral orbitofrontal cortex (OFC) (x = 21, y = 47, z = 4, k = 19, T = 4.67), the inferior frontal gyrus (x = 30, y = 44, z = 4, k = 29, T = 4.71) and the nucleus accumbens (x = 24, y = 32, z = − 2, k = 14, T = 4.71) after Ket + LSD treatment (p < 0.05 FWE corrected after SVC; Fig. 3A). Furthermore, comparing the LC > NC FB contrast revealed a significant increase in BOLD signal in the mPFC (x = − 6, y = 65, z = 25, k = 20, T = 3.42) and the inferior frontal gyrus (x = 30, y = 41, z = 4, k = 74, T = 3.99) after LSD treatment (p < 0.05 FWE corrected after SVC; Fig. 3B). For the HC > NC R2 contrast, the analysis revealed a significantly higher BOLD signal in the in the medial OFC cortex (x = 3 y = 47, z = 4, k = 123, T = 4.67) and the lateral OFC (x = 12, y = 41, z = − 2, k = 15, T = 3.82) in the Ket + LSD condition (Fig. 3C).

##### Ket + LSD vs. Placebo condition

No significant differences in any contrast were found when comparing Ket + LSD and Pla treatment conditions (p < 0.05 FWE corrected after SVC).

#### Correlations between personality traits and changes in adaptation to social norms

**Figure 4:**
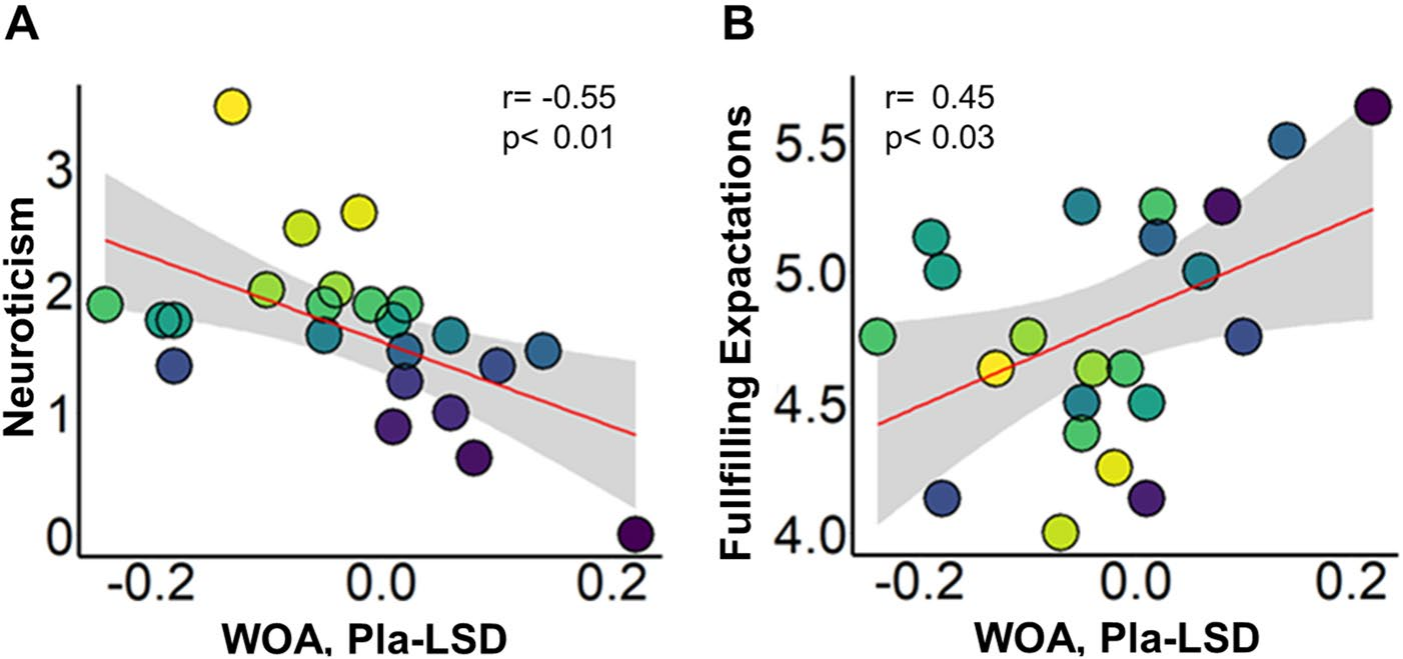
Correlations between personality traits and changes in adaptation to social norms. (**A**) Negative association between the Big Five personality trait Neuroticism and the LSD-induced change in WOA score (Pla-LSD, LC and HC conditions) (r = − 0.55, p < 0.01). (**B**) Positive association between the personality trait Fulfilling Expectations and the LSD-induced change in WOA score (Pla-LSD, LC and HC conditions) (r = 0.45, p < 0.03). Data points are color coded for each individual and rank-ordered according to their Neuroticism score (**A**). Grey background in scatterplots indicates the 95% confidence interval. *n* = 24.

A significant and negative Pearson correlation was found between the Neuroticism-Extraversion-Openness Five-Factor Inventory (NEO-FFI) Big Five personality trait “Neuroticism” and the LSD-induced change social adaptation (WOA score Pla-LSD) (r = − 0.55, p < 0.01; Fig. 4A). Additionally, a positive relationship was found between the Social Responsibility Scale (SRS) subscale “Fulfilling Expectations” and the change in WOA score (Pla-LSD) (r = 0.450, p < 0.03; Fig. 4B). The personality traits Neuroticism and Fulfilling Expectations were negatively correlated (r = − 0.455, p < 0.03). Additionally, we investigated correlations between beta values (see Fig. 2E) and personality traits as well as WOA scores within each treatment condition. No significant correlations were found.

## Discussion

This study presents strong evidence for the impact of LSD on social influence processing and social decision-making and its underlying neuropharmacology by combining pharmacological manipulations with behavioral and neuroimaging methods. Our results show that (1) LSD increases the adaptation to the opinion of others, but only if they are similar to one’s own. This was associated with BOLD signal changes in the mPFC. (2) LSD modulates the mPFC in particular during social feedback processing. (3) Social feedback processing may be dependent on the 5-HT_2A_ receptor system as LSD-induced effects were blocked by ketanserin, while social decision-making may be modulated by other receptors. (4) The magnitude of LSD-induced changes in social adaptation is associated with personality factors.

### LSD increases social adaptation to opinions similar to one’s own

In line with previous studies^4,13^ processing highly conflicting social feedback was associated with increased BOLD signal in the mPFC, SMA, ventral striatum, and the precuneus under placebo. Differences in behavioral adaptation towards the group norm between high and low conflict conditions did not reach statistical significance in the Pla condition. However, in the LSD condition participants showed an increased adaptation to the group norm in the LC compared to the HC condition. When comparing the BOLD response between treatment conditions, the BOLD signal was increased in the mPFC in response to HC vs. LC in the Pla condition. In contrast, in the LSD condition BOLD signal in the mPFC was increased in response to LC feedback compared to NC, but not in the HC condition. These results are in line with a previous study^32^ reporting that LSD increases suggestibility, but extends these findings by showing that LSD does not induce general increases in social adaptation, but specifically intensifies adaptation to opinions that are similar to the individual’s own.

The reason for this may be that LSD induces changes in self-relevance processing^29^, potentially leading to an increase in assigned value to opinions which are not in high conflict. At the same time, the need to adapt to avoid social punishment may be diminished in the LSD condition^31^ explaining a lower adaptation when opinions are in high conflict. Several studies have linked the mPFC to behavior change during exposure to group norm or persuasive messages and posited that this relationship is due to self-related processing via subjective value assignment^35,36^. Furthermore, previous studies have identified the mPFC as a key region for the integration of reward-, self-, and mentalizing processes during social feedback processing^37^. The integration of information about value in relation to the self has been suggested to be key for attitude or behavior change^36^. Yet, the mPFC cortex has also been associated with salience processing^38^. However, it has been shown that the mPFC consists of several anatomically and functionally distinct regions^39^. While salience processing has mostly been associated with regions such as the dorsal anterior cingulate cortex^38^, the anterior mPFC—overlapping with the regions showing changes in BOLD signal under LSD in this study—has been associated with feedback-related surprise^39^. As previous findings have shown that LSD alters sensitivity to surprising stimuli^40,41^ future studies are needed to investigate whether the effects observed here are specific to social feedback processing or also occur in a non-social context.

Furthermore, the anterior part of the mPFC has been associated with assigning personal significance to stimuli^42^. The serotonin system has been implicated in value-based decision-making^43^. Importantly, LSD has been shown to induce alterations in self-relevance processing^29,44^ and a LSD-induced loosening of self-other boundaries was associated with changes in social interaction^45^. We therefore propose that LSD alters the computation of value assigned to a group norm via increased processing of self-relevance in the mPFC when participants are exposed to opinions not conflicting too much with their own. When opinions of others are similar to one’s own they may be processed as more valuable and more self-relevant due to LSD-induced loosening of self-other boundaries. This may lead to a subsequent behavior change towards more adaptation to the group norm when social conflict is low.

In the HC condition however, the perception of the discrepancy between one’s own and the group norm may cause cognitive dissonance^46^. The dissonance induced by strong disagreement with the group norm elicits a negative tension state and may induce feelings of social rejection^47^. The avoidance of social punishment is one of the main motives for humans to be socially conform^48^. Participants who are more sensitive to social exclusion are more likely to adapt their behavior to fit in with others^49^. Furthermore, it has been shown that avoidance of social punishment has an effect on conformity behavior even in the absence of a social punishment stimulus^50^. This may explain why participants adapt their opinion more strongly to the group norm in the placebo condition when exposed to high conflict. However, the motivation to avoid social rejection or punishment may be diminished in the LSD condition because other studies have linked stimulation of the 5-HT_2A_ receptor to reduced reaction to social exclusion and social pain^31^. Therefore, the need to conform to avoid social punishment may have been reduced in the LSD condition leading to less adaptation when exposed to highly conflicting opinions.

### LSD modulates the mPFC during social feedback processing

The paradigm employed in this study allowed to measure changes in BOLD signal during two time points associated with different cognitive processes: (1) while participants received social feedback and (2) when they subsequently rated the stimuli (social decision-making). Our fMRI data reveal that LSD-induced changes in BOLD signal in the mPFC in the LC condition occurred during social feedback processing and not during social decision-making. Therefore, LSD seems to increase adaptation to other persons’ opinions due to alterations in feedback processing rather than social decision-making. This is in line with our interpretation outlined above suggesting that LSD changes the value assigned to the opinions of others via alterations in self-relevance processing associated with alterations in activity in the mPFC.

### Social feedback processing may depend on the 5-HT_2A_receptor system

LSD-induced changes in the mPFC during social feedback processing were fully blocked by the 5-HT_2A_ receptor antagonist ketanserin. Furthermore, no differences in BOLD signal were found between Pla and Ket + LSD during feedback processing. This indicates that LSD-induced changes in social feedback processing depend on the 5-HT_2A_ receptor system.

Regarding social decision-making, our fMRI data showed that Ket + LSD compared to LSD increased the BOLD signal in the medial OFC extending into the mPFC and the lateral OFC. Ketanserin blocked LSD-induced 5-HT_2A_ receptor stimulation, however LSD has affinity at various serotonin and dopamine receptors^28^ and animal studies showed that dopamine receptor stimulation may contribute to the effects of LSD^26^. It is therefore conceivable that these changes in brain activity during social decision-making are modulated via dopamine rather than 5-HT systems, in particular since previous results have implicated the dopamine system in decision-making processes^51^. Our results are also in line with previous findings showing that decision-making is a cognitive domain not influenced by stimulating the 5-HT_2A_ receptor^52^. Therefore, social feedback processing and socially influenced decision-making are potentially modulated by two different neurotransmitter systems, i.e. the 5-HT_2A_ and potentially the dopamine system respectively.

### The magnitude of LSD-induced changes in social adaptation is associated with personality

Our results show a negative correlation between the Big Five personality trait Neuroticism and LSD-induced adaptation to social norms. Neuroticism is characterized by a tendency to experience negative affect and is associated with psychiatric diseases such as depression and anxiety disorders^53^. Participants with higher scores in Neuroticism show a greater change from Pla, i.e. they adapt more strongly after LSD administration. As outlined above, stimulation of the 5-HT_2A_ receptor has been shown to attenuate negative emotion and fear processing, while at the same time enhancing empathy^54^, heightening positive mood^55^ and leading to increases in the personality trait openness^55–57^. LSD may therefore have a particularly strong impact on participants with a tendency to negative and anxious affect, perhaps by reducing these feelings and rendering them more open to social interaction and the opinions of others. Yet, we show that LSD increases social adaptation in particular when the conflict with the group norm is low, and that reduced rejection sensitivity may prevent participants from adapting their opinions to strongly differing group norms. However, in participants with high baseline neuroticism and anxiety this latter effect may be diminished or not strong enough to overcome fear of rejection resulting in increased adaptation across conflict conditions.

Furthermore, we found a positive correlation between the personality trait “Fulfilling Expectations” and LSD-induced changes in social adaptation. Participants with lower scores adapted more strongly after LSD. Additionally, “Fulfilling Expectations” was negatively correlated with Neuroticism. It is therefore conceivable that LSD has the strongest impact on social cognition in participants who show low sociability and high Neuroticism before administration, potentially by increasing empathy and decreasing anxiety. However, given the exploratory nature of this analysis, these results need replication in future studies.

## Limitations

This study provides empirical evidence for the involvement of the 5-HT_2A_ receptor system in social feedback processing while social decision-making may be modulated by different receptor systems. However, other receptors stimulated by LSD have not been blocked in this study. Therefore, no conclusions can be drawn about the impact of other receptors on LSD-induced effects. Furthermore, the current study did not investigate the effect of ketanserin without LSD on social influence processing and decision-making. A full design should be employed in future studies. A further limitation is the lack of a non-social control condition. Future studies should include this non-social control to investigate if the results are specific for social feedback or represent a more global alteration of conflict or error processing.

## Conclusion

In sum, LSD increased social adaptation to group opinions that are relatively similar to one’s own via alterations in social feedback processing associated with stimulation of 5-HT_2A_ receptors and increased activity of the mPFC. While social feedback processing seems to depend on the 5-HT_2A_ receptor system, social decision-making may engage other neurotransmitter systems such as the dopamine system. We are constantly exposed to social norms and opinions that shape our daily decisions and are vital for successful social interactions and communication. In light of the profound impact of social interactions on physical and mental health, it is, therefore, crucial to have a better neurobiological understanding of these processes given that psychotherapy and various medications used in psychiatry target these cognitive functions. Furthermore, these results may have important implications for psychedelic-assisted therapeutic approaches currently being evaluated in various clinical trials.

## Materials and methods

### Participants

Participants were recruited through advertisements placed at local universities and underwent a screening visit before inclusion into the study. All included subjects were healthy according to medical history, physical examination, blood analysis, and electrocardiography and had normal or corrected-to-normal vision. The Mini-International Neuropsychiatric Interview (MINI)^58^, the Diagnostic and Statistical Manual of Mental Disorders, fourth edition self-rating questionnaire for Axis-II personality disorders (SCID-II)^59^, and the Hopkins Symptom Checklist (SCL-90-R)^60^ were used to exclude subjects with present or previous psychiatric disorders or a history of major psychiatric disorders in first-degree relatives. Volunteers were included when they were between 20 and 40 years of age and willing to abstain from the use of any prescription or illicit drug for a minimum of 2 weeks before the first test day and for the duration of the entire study, and to abstain from drinking alcohol for at least 24 h before test days. Urine tests were used to verify the absence of drug and alcohol use and to exclude pregnancy. Participants were furthermore required to abstain from drinking coffee during the test day and to abstain from smoking for at least 60 min before MRI assessment. Further exclusion criteria included cardiovascular disease, history of head injury or neurological disorders, history of alcohol or drug dependence or abuse, left-handedness, poor knowledge of the German language, MRI exclusion criteria including claustrophobia, and previous significant adverse reactions to a hallucinogenic drug.

The initial study population consisted of 25 participants. One participant was excluded due to excessive head movement during scanning (> 3 mm) at the third assessment (Pla). Therefore, the final sample consisted of 24 participants (*n* = 18 males and 6 females; mean age (*M*) = 25.25 years; standard deviation (*SD*) = 3.72 years) which were included in the statistical analyses. Before participating, all participants provided written informed consent after having received detailed written and oral descriptions of the study procedures, as well as details regarding the effects and possible risks of the substances administered in accordance with the declaration of Helsinki. The current data were collected as part of a larger study^29, 45^. The study was approved by the Cantonal Ethics Committee of Zurich (KEK) and the Swiss Federal Office of Public Health (BAG), Bern, Switzerland, authorized the use of LSD in humans. The study was registered at clinicaltrials.gov (NCT02451072).

No substantial side effects were recorded during the study. Four participants reported transient mild headaches after drug effects had worn off. One participant reported transient sleep disturbances for the first two nights after drug administration. Participants were contacted again three months after the last drug administration. No further side effects were recorded.

### Study design and procedure

At the screening visit participants completed the NEO-FFI^61^ and the SRS^62^ to assess personality traits. The Big Five personality traits *Extraversion *(*M* = 3.81, *SD* = 0.52), *Agreeableness* (*M* = 3.92, *SD* = 0.76), *Conscientiousness* (*M* = 4.01, *SD* = 0.84), *Neuroticism* (*M* = 1.66, *SD* = 0.68) and *Openness to Experience* (*M* = 4.01, *SD* = 0.74) as well as the SRS questionnaire which comprises two subscales *fulfilling expectations* (*M* = 4.80, *SD* = 0.44) and *adherence to social rules* (*M* = 3.89, *SD* = 0.58) were assessed. This study employed a double-blind, randomized, placebo-controlled, within-subject design with three experimental sessions, each separated by at least 2 weeks. At the beginning of each experimental session before drug administration a urine test for drug-screening and pregnancy test was conducted. Each participant received either:

1. placebo + placebo (Pla) condition: placebo (179 mg Mannitol and Aerosil 1 mg po) after pretreatment with placebo (179 mg Mannitol and Aerosil 1 mg po)
2. placebo + LSD (LSD) condition: LSD (100 μg po) after pretreatment with placebo (179 mg Mannitol and Aerosil 1 mg po)
3. ketanserin + LSD (Ket + LSD) condition: LSD (100 μg po) after pretreatment with the 5-HT2A antagonist ketanserin (40 mg po).

60 min after pretreatment with the first capsule (placebo or ketanserin) the second one (placebo or LSD) was administered. The social influence paradigm was conducted 330 min after second drug administration while undergoing the MRI acquisition. To assess the subjective experience after drug intake, the 5D-ASC (a retrospective self-report questionnaire)^63^ was administered to participants 720 min after drug treatment. Mood state was assessed using the PANAS^64^. Participants completed the PANAS 10 min before pretreatment with placebo or ketanserin and 720 min after treatment with placebo or LSD to retrospectively rate mood state at the time of peak subjective effects. For details and assessment of subjective effects, see Supplementary Methods.

### Social influence paradigm

**Figure 5:**
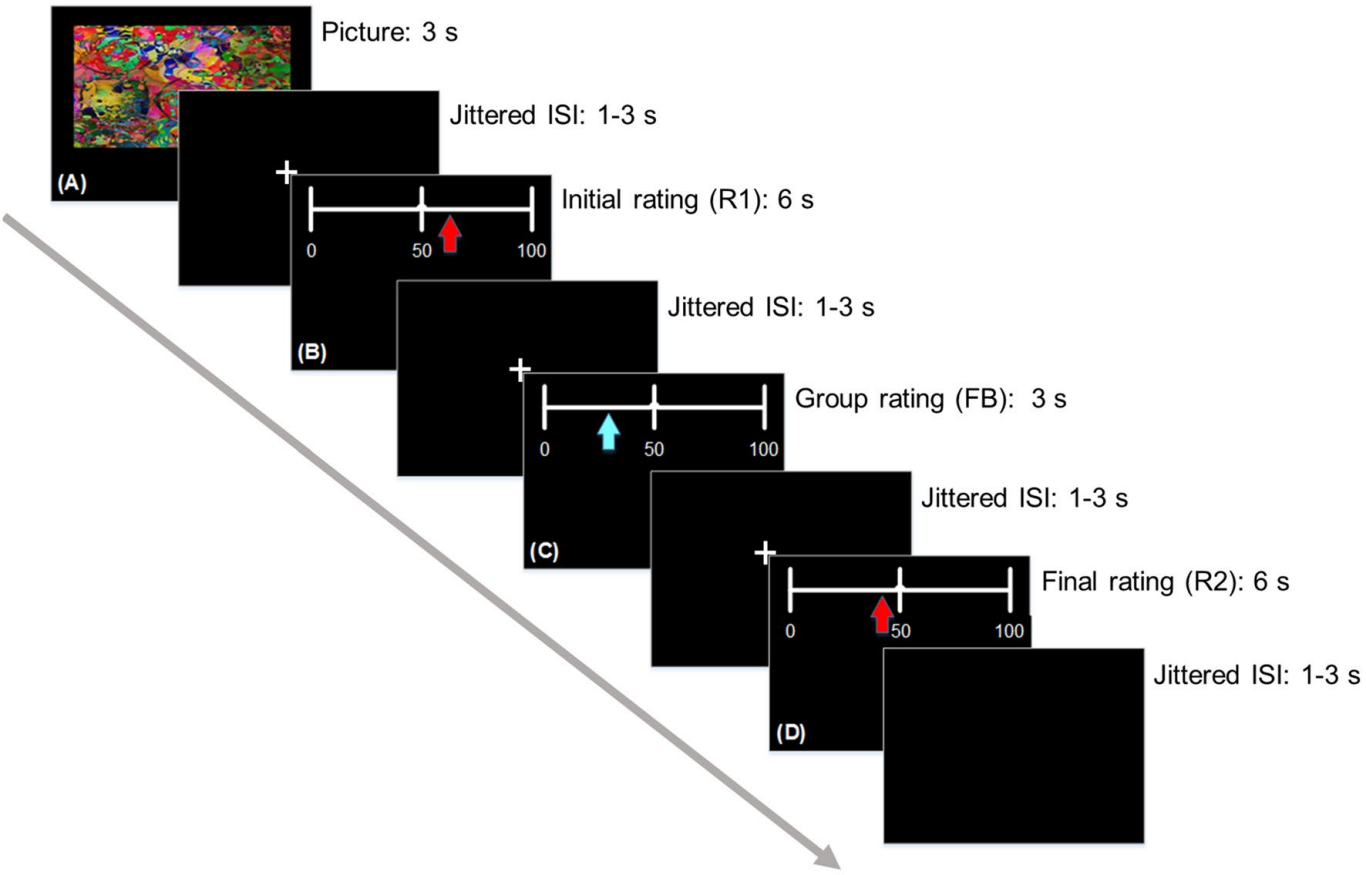
Example trial of the social influence paradigm. (**A**) Presentation of a picture of street art. (**B**) Participants give their first rating (R1) indicating their perceived aesthetic quality of the picture on a scale from 0 to 100. (**C**) Presentation of the group rating (FB) which represents the "social norm". Subjects are instructed that this score represents the average value of the rating of this picture collected from 70 other participants. (**D**) Participants are requested to rate the picture again in the final rating (R2). They can either confirm or change their initial rating.

The Social Influence Paradigm (SIP) was adapted from Schilbach et al.^13^. All participants received standardized instructions and were familiarized with the task in an exercise trial before drug administration. Participants were asked to rate pictures of artwork which were selected and grouped into three matched parallel versions according to a pre-study survey (see Supplementary Methods). We decided to use pictures of street art as stimulus material for the SIP since the perception of aesthetic quality is inherently subjective and does not require skills (e.g., arithmetic calculations) that may be influenced by expertise (i.e., there is no right or wrong answer) or pharmacological manipulation. While undergoing fMRI scanning, participants rated the stimuli according to their perceived aesthetic quality by moving an arrow on a screen on a scale from 0 (“not at all”) to 100 (“high”) and pressing a button when the arrow reached the intended position. After this initial are presented with the feedback (FB) of the group rating representing the “social norm”. This feedback of the group rating was presented on the same scale as the participants ratings. Participants were told that the group rating represents the average rating of 70 previous participants collected in a different experiment. After the presentation of the FB subjects were required to rate their pictures again (final rating, R2) (Fig. 5). The pictures were each presented for three seconds and the subject was required to provide their rating by pressing the button on a MRI-compatible response mouse pad within 6 s after the picture/group rating presentation. Between these events (R1, FB and R2) a jittered Inter-Stimulus Interval (ISI) of 1–3 s (mean 2 s) was presented showing a fixation cross. Unknown to participants, the average rating of the group norm varied algorithmically as a function of the initial rating provided by the participant to ensure the occurrence of social conflict. By subtracting R1 from the group norm the paradigm generated three different conditions:

$$\begin{aligned} & {\text{No conflict }}\left( {{\text{NC}}} \right) \, \left( {0 - {5 } \pm } \right) \\ & {\text{Low conflict }}\left( {{\text{LC}}} \right) \, \left( {{15} - {25 } \pm } \right) \\ & {\text{High conflict }}\left( {{\text{HC}}} \right) \, \left( { > {65 } \pm } \right) \\ \end{aligned}$$

Each social conflict condition was presented 20 times per session in a pseudo-randomized order. In total, each subject completed 60 trials per session. Participants completed two successive runs comprising 30 trials each. The duration of each run was approximately 15 min. As described above, three parallel versions were developed and administered in a counter-balanced randomized order to the subject across experimental days.

All visual stimuli were presented using the software package Presentation (Version 17.0: Neurobehavioral Systems, Albany, CA, USA) and were presented with MRI-compatible video googles (NordicNeuroLab Visual System). For details of MRI data acquisition and preprocessing, see Supplementary Methods.

### Statistical analysis of social influence paradigm

#### Rating 1

The initial ratings (R1) were analyzed by using a repeated-measures ANOVA with R1 and treatment condition as within-subject factors followed by simple main effect analyses and Bonferroni-corrected pairwise comparisons.

#### Weight of advice (WOA)

The WOA Score represents the adaptation to the group norm^13^. The WOA score is defined as the absolute value of the change between initial (R1) and final judgment (R2) in relation to the absolute distance between the initial judgment (R1) and final group rating (FB) for each trial:

$${\text{WOA}} = {\text{abs}}\left( {{{\left( {{\text{final judgment }}\left( {{\text{R2}}} \right){-}{\text{initial judgment }}\left( {{\text{R1}}} \right)} \right)} \mathord{\left/ {\vphantom {{\left( {{\text{final judgment }}\left( {{\text{R2}}} \right){-}{\text{initial judgment }}\left( {{\text{R1}}} \right)} \right)} {\left( {{\text{group rating }}\left( {{\text{FB}}} \right){-}{\text{initial judgment }}\left( {{\text{R1}}} \right)} \right)}}} \right. \kern-\nulldelimiterspace} {\left( {{\text{group rating }}\left( {{\text{FB}}} \right){-}{\text{initial judgment }}\left( {{\text{R1}}} \right)} \right)}}} \right)$$

The WOA assumes a value of 0 when final and initial judgment are identical, i.e. when the subject confirms his initial rating (R1) in the second rating (R2) by ignoring the group rating (FB). The WOA value becomes 1 if the judge completely adjusts to the group norm and > 1 when the final judgment exceeds the group norm. The WOA is calculated form the ratings provided by the participants while conducting the SIP during fMRI scanning. Treatment (Pla, LSD, Ket + LSD) and condition (LC, HC) were entered as within-subject factors in a repeated measures ANOVA to investigate their influence on WOA values. Significant main effects and interactions were followed by Bonferroni-corrected pairwise comparisons and simple main effect analyses.

#### fMRI data

Picture presentations, R1, FB and R2 were modeled as blocks with the following durations: 3 s, 6 s, 3 s, 6 s, respectively and convolved with a canonical hemodynamic response function in the first-level analysis for each subject. Low frequency drifts were filtered with a 128 s high pass filter. FB and R2 events were divided into the following conditions for each participant: NC, LC, and HC. The following contrasts were computed for each participant and treatment condition for FB and R2: (1) HC > LC, (2) HC > NC, (3) LC > NC. To identify brain regions sensitive to social conflict, we conducted a second level group analysis investigating these contrasts in the Pla condition using one-sample t-tests. To investigate the influence of our pharmacological intervention, we compared these contrasts between the treatment conditions LSD > Pla, LSD > Ket + LSD, and Ket + LSD > Pla using paired t-tests according to a summary statistics approach. Due to our a priori hypothesis, we were interested in areas sensitive to social influence processing. Therefore, we defined five ROIs previously identified to be involved in social influence processing that were then used for small volume correction (SVC) in our analyses 2011^4^: mPFC (x = − 4, y = 56, z = 28), lateral OFC (x = 18, y = 48, z = − 4), inferior frontal gyrus (x = 38, y = 44, z = 8), right nucleus accumbens (x = 22, y = 22, z = − 2), SMA (x = 14, y = 26, z = 52) and precuneus (x = 4, y = − 62 z = 38). Search volumes were defined as spheres with a 10-mm radius centered on these previously reported MNI coordinates^4^. Peak-level familywise error corrections (FWE) were applied in the SVC ROI analysis at a threshold of p < 0.05, k > 10 (Table S2). All brain coordinates are reported in the MNI atlas space.

#### Correlation analyses

To further investigate the relationship between personality traits and the adaptation to the group norm assessed with the WOA score, Pearson correlation analyses between the WOA score (Pla-LSD change score, mean of LSD and HC conditions) and the NEO-FFI Big Five personality traits ”Extraversion”, “Agreeableness”, “Conscientiousness”, “Neuroticism” and “Openness to Experience”, as well as the SRS subscales “Fulfilling Expectations” and “Adherence to Social Rules” and the WOA score were computed in an exploratory analysis. Results were therefore not corrected for multiple comparisons.

## Supplementary information

Supplementary information
