# Supplementary material for: LSD-induced increases in social adaptation to opinions similar to one’s own are associated with stimulation of serotonin receptors
Source: Sci Rep. 2020 Jul 22;10:12181. doi: 10.1038/s41598-020-68899-y (PMC7376162; doi:10.1038/s41598-020-68899-y)
Supplement: Supplementary file 1 — Supplementary information [file 41598_2020_68899_MOESM1_ESM.docx]

**Supplementary Material**

**LSD-induced increases in social adaptation to opinions similar to one’s own are associated with stimulation of serotonin receptors**

*Patricia Duerler, Msc^a^; Leonhard Schilbach^b,c,d^, MD; Philipp Stämpfli^e^, PhD; Franz X. Vollenweider^a^, MD; Katrin H. Preller^a^, PhD

***Corresponding author:**

Patricia Duerler, MSc

E-mail: patricia.duerler@bli.uzh.ch

**This PDF file includes:**

Supplementary Methods

Supplementary Results

Figures S1 to S4

Tables S1 to S3

Supplementary References

**Supplementary Methods**

**Pre-study survey**

For stimuli selection, a pre-study survey was conducted to ensure eligible and homogeneous stimulus material, and to create matched stimuli sets comprising the three parallel-version for each experimental test day.

*Participants:* This study included 69 participants aged between 19 to 64 years (27 males, 42 females; *M* = 31.5 years, *SD* = 8.7 years).

*Procedure:* The survey for the evaluation of the pictures was accessible via an online survey tool (www.findmind.ch). Participants were recruited via mailing lists and did not take part in the main study. A set of 200 pictures of street art was composed. Pictures with illustrations of real persons, writings or letters were not included. Only painted pictures which evoked as little associations as possible with real places, persons, or situations were presented. The pictures were randomly subdivided into three subsets each comprising 66 and 67 pictures which were randomly distributed to the 69 participants (*n* = 23 for the evaluation of each set). The criteria for the inclusion of pictures for the Social Influence Paradigm (SIP) was the neutrality of the presented content to ensure the selection of an eligible artwork and the development of matched parallel versions for the three testing days. To assess the impact of the pictures on perception participants had to rate each picture upon three criteria: their perceived “*aesthetic quality* ("How aesthetically pleasing do you find the picture?"), *emotional arousal* ("How strongly does the picture touch you emotionally?") and “*deeper meaning”* ("Does the picture have a deeper meaning for you?") upon a scale from one to four (1: not at all; 4: very much). Pictures with extreme values (>3) in the evaluation of the three criteria were excluded to ensure homogenous stimulus material. For the SIP, 180 pictures were included and divided in three matched sets for the experimental testing days (60 per session) which were administered in a randomized counterbalanced fashion across testing days.

*Statistical analyses:* To investigate if these three sets differ in their ratings a multivariate factorial ANOVA with three dependent variables for the ratings “emotional arousal”, “aesthetic quality” and “deeper meaning” and the factor “set” was conducted in SPSS 23 (IBM, 2012). The significance threshold was set at p < 0.05 (two-tailed). Greenhouse-Geisser and Bonferroni correction for p-values were utilized if necessary.

*Results:* There were no significant differences between the three sets for the ratings for emotional arousal (F(2, 177) = 2.25, p > 0.1), aesthetic quality (F(2, 177) = 2.61, p > 0.07) an deeper meaning (F(2, 177) = 2.54, p > 0.08). The three sets were therefore well matched for emotional arousal, aesthetic quality, and deeper meaning (**Table** **S3**).

#### MRI data acquisition and preprocessing

Magnetic resonance data were acquired on a Philips Achieva 3.0T whole-body scanner (Best, The Netherlands). A 32-channel receive head coil and MultiTransmit parallel radio frequency transmission was used. Images were acquired using a whole-brain, gradient-echo planar imaging (EPI) sequence (repetition time, 2,500 ms; echo time, 27 ms; slice thickness, 3 mm; 45 axial slices; no slice gap; field of view, 240 × 240 mm^2^; in-plane resolution, 3 × 3 mm^2^; sensitivity-encoding reduction factor, 2.0). Additionally, high-resolution anatomical images (voxel size, 0.7×0.7×0.7 mm^3^) were acquired by using a standard T1-weighted 3D magnetization prepared rapid-acquisition with gradient echo (MPRAGE) sequence. All relevant data are available from the authors. Images were analyzed using SPM12 (https://www.fil.ion.ucl.uk). The preprocessing steps consisted of slice time correction, realignment, spatial normalization to the standard EPI template of the Montreal Neurological Institute (MNI), and spatial smoothing using a Gaussian kernel of 8-mm full-width half-maximum (FWHM) to meet the statistical requirements of the general linear model (GLM).

**Statistical analysis of subjective effects**

Data were analyzed using IBM SPSS Statistics 23 software (IBM, Chicago, Illinois, USA). The 5D-ASC questionnaire contains 94 items to be answered on visual analogue scales[1][^⁠⁠^](#_CTVL001b2b940533c334222ba27f8f43161708d). Ratings were calculated for 11 validated scales[[2]](#_CTVL001cf43b97d62784449956cb02b7ee4d301): *experience of unity*, *spiritual experience*, *blissful state*, *insightfulness*, *disembodiment*, *impaired control and cognition*, *anxiety*, *complex imagery*, *elementary imagery*, *audio-visual synesthesia*, and *changed meaning of percepts*. For the 5D-ASC ratings a repeated-measures ANOVA with treatment condition (Pla, LSD, and Ket+LSD) and scale as within-subject factors was computed. For the PANAS[[3]](#_CTVL001c6efcb5b737d47ae86b58a9b44d39e23) ratings, scores were calculated for the *positive* and *negative* affect scales and analyzed using a repeated-measures ANOVA with treatment condition (Pla, LSD, and Ket+LSD), time (pre-drug administration and post-drug administration), and scale (positive affect and negative affect) as within-subject factors. Bonferroni-corrected pairwise comparisons and simple main effects analyses followed significant main effects or interactions. Statistical comparisons of all data were carried out with a significance level of p < 0.05 (two-tailed).

**Supplementary Results**

**Subjective drug effects**

#### For the investigation of the subjective drug effects assessed with the retrospectively administered 5D-ASC questionnaire a repeated measures ANOVA (treatment * scale) was conducted. There was a significant main effect for treatment (F(2,46) = 90.12, p < 0.001) and scale (F(10, 230) = 15.76, p < 0.001) and a significant treatment x scale interaction (F(20, 460) = 14.210, p < 0.001). Bonferroni corrected simple main effect analyses showed that LSD significantly increased all 5D-ASC scale scores compared with Pla and Ket+LSD conditions (all p < 0.001) except for spiritual experience and anxiety (all p > 0.20). There were no significant differences between Pla and LSD+Ket scores (all p > 0.90; Fig S1). All LSD-induced subjective drug effects were blocked by ketanserin.

**Subjective drug effects on mood state**

A repeated-measures ANOVA (time * treatment * scale) for the PANAS revealed a significant main effect for scale (F(1, 23) = 232.13, p < 0.001), revealing higher scores on the positive affect scale than on the negative affect scale, and a significant main effect for treatment (F(2, 46) = 6.23, p < 0.01). Furthermore, significant interactions were found for time * treatment (F(2, 46) = 20.70, p < 0.001), time * scale (F(1, 23) = 11.11, p < 0.01), and time * treatment * scale (F(2, 46)= 3.08, p < 0.05). Bonferroni corrected simple main effect analyses revealed that scores on the positive and negative affect scales did not differ between the treatment conditions before drug administration (all p > 0.9). After drug administration, there was a significant difference in the LSD treatment condition indicating heightened positive and negative affect compared to the Pla and Ket+LSD conditions (all p < 0.05). Scores in the Pla und Ket+LSD treatment conditions did not differ for either positive nor negative affect scales (all p > 0.9; **Fig. S2**).

**Supplementary Figures**


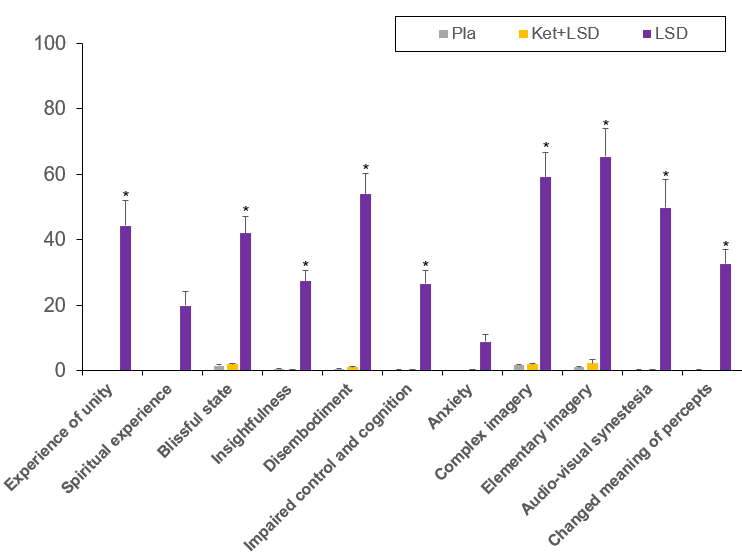


**Fig. S1.** **Subjective drug effects.** Retrospectively assessed with the 5D-ASC questionnaire 720 min after each drug treatment. Scores are expressed as a percent of the scale maximum. LSD significantly increased all scale scores compared to Pla and Ket+LSD except for spiritual experience and anxiety. LSD-induced subjective drug effects were blocked by ketanserin. Data are expressed as mean and standard error of the mean. *p < 0.05, Bonferroni corrected, *n* = 24 participants.

**
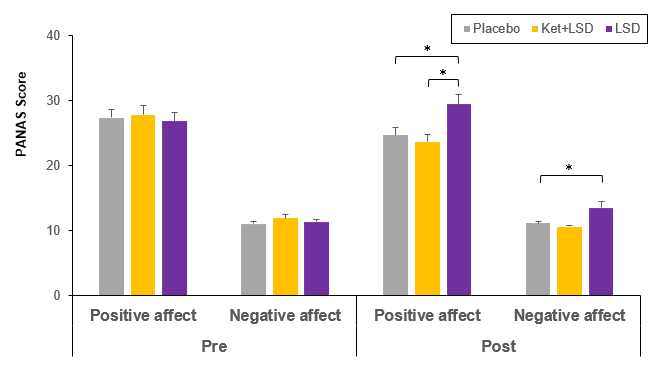
Fig. S2.** **Subjective drug effects on mood state**. Ratings on the Positive and Negative Affect Schedule (PANAS) in the Pla, Ket+LSD, and LSD treatment conditions. The questionnaire was completed 10 minutes before each treatment (pre) to assess current mood state and 720 minutes after each drug treatment (post) to asses peak drug effects retrospectively. Data are displayed as mean and standard error of the mean. *p < 0.05, Bonferroni corrected, *n* =24.

**
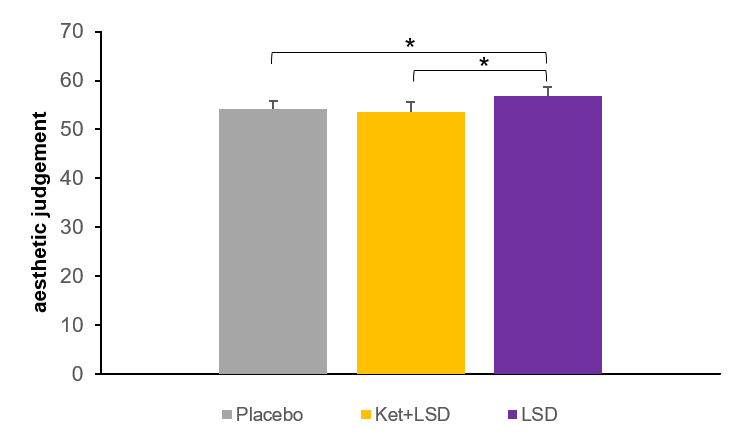
**

**Fig. S3.** **Rating 1.** Aesthetic judgements derived from the first rating in the SIP (scale from 0-100). Comparing the first rating between the treatment conditions Pla, Ket+LSD and LSD revealed a significant difference between the LSD and Pla, and the LSD and Ket+LSD condition. Data are displayed as mean and standard error of the mean. *p < 0.05, Bonferroni corrected, *n* = 24.

**
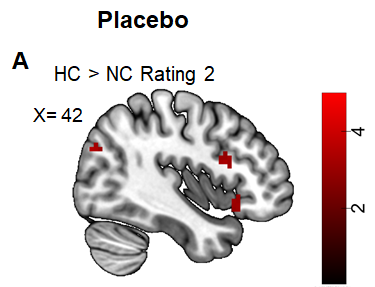
**

**Fig. S4. fMRI Data in the Placebo treatment condition for the final judgment (R2)** Differences in BOLD signal for (**A**) HC > NC Rating 2 contrast at peak in the angular gyrus (x= 42 , y= -79, z= 28); p < 0.05 FWE corrected (peak level after SVC); data displayed at p < 0.005, k > 10 (uncorrected). *n* = 24.

**Supplementary Tables**

**Table S1. WOA score means and standard error of the means for treatment conditions and post hoc comparisons with effect sizes (Cohen’s d)**

| ***Condition*** | **Mean** | **SD** |  |
| --- | --- | --- | --- |
|  |  |  |  |
| **Pla** |  |  |  |
| LC | .19 | .10 |  |
| HC | .25 | .14 |  |
| **LSD** |  |  |  |
| LC | .27 | .14 |  |
| HC | .21 | .12 |  |
| **Ket+LSD** |  |  |  |
| LC | .21 | .11 |  |
| HC | .23 | .14 |  |
|  |  |  |  |
| **Post hoc comparisons** | **Mean** | **SD** | **Effect size *d*** |
|  |  |  |  |
| **Within treatment conditions** | |  |  |
| Pla LC vs. Pla HC | -.05 | .16 | -0.34 |
| LSD LC vs. LSD HC | .06 | .13 | 0.46 |
| Ket+LSD LC vs. Ket+LSD HC | -.01 | .10 | -0.11 |
|  |  |  |  |
| **Between treatment conditions** | |  |  |
| Pla LC vs. LSD LC | -.08 | .11 | -0.70 |
| Pla LC vs. LSD HC | -.02 | .11 | -0.19 |
| Pla LC vs. Ket+LSD LC | -.02 | .10 | -0.21 |
| Pla LC vs. Ket+LSD HC | -.03 | .14 | -0.23 |
| Pla HC vs. LSD LC | -.02 | .17 | -0.14 |
| Pla HC vs. LSD HC | .03 | .19 | 0.18 |
| Pla HC vs. Ket+LSD LC | .03 | .18 | 0.19 |
| Pla HC vs. Ket+LSD HC | .02 | .20 | 0.11 |
| LSD LC vs. Ket+LSD LC | .06 | .13 | 0.45 |
| LSD LC vs. Ket+LSD HC | .05 | .17 | 0.28 |
| LSD HC vs Ket+LSD LC | .00 | .10 | 0.00 |
| LSD HC vs Ket+LSD HC | -.01 | .12 | -0.09 |

HC, high conflict; LC, low conflict; NC, no conflict; FB; Feedback processing; R2, final judgment; Pla, placebo; Ket, Ketanserin; Effect size *d* (Cohen’s *d*), *n* = 24

**Table S2. BOLD signal changes**

|  | **Brain Region** | **x** | **y** | **z** | **k** | **T** |
| --- | --- | --- | --- | --- | --- | --- |
| **Pla** |  |  |  |  |  |  |
| *HC > LC FB* |  |  |  |  |  |  |
|  | Medial prefrontal cortex | -3 | 47 | 25 | 70 | 3.74* |
|  | Supplementary motor area | 9 | 20 | 49 | 44 | 4.61* |
|  | Nucleus accumbens | 30 | 23 | -5 | 15 | 4.12* |
|  | Precuneus | 0 | -70 | 40 | 10 | 3.23* |
| *HC > NC FB* |  |  |  |  |  |  |
|  | Supplementary motor area | 6 | 29 | 52 | 36 | 4.54* |
| *HC > NC R2* |  |  |  |  |  |  |
|  | Angular gyrus | 42 | -79 | 28 | 10 | 3.49* |
| **Pla > LSD** |  |  |  |  |  |  |
| *HC > LC*  *FB* |  |  |  |  |  |  |
|  | Medial prefrontal cortex | 0 | 59 | 19 | 32 | 3.93* |
| **LSD > Pla** |  |  |  |  |  |  |
| *LC > NC FB* |  |  |  |  |  |  |
|  | Medial prefrontal cortex | -9 | 62 | 25 | 34 | 3.12* |
| **LSD > Ket+LSD** | |  |  |  |  |  |
| *HC > NC FB* |  |  |  |  |  |  |
|  | Lateral orbitofrontal cortex | 21 | 47 | 4 | 19 | 4.67* |
|  | Inferior frontal gyrus | 30 | 44 | 4 | 29 | 4.71* |
|  | Nucleus accumbens | 24 | 32 | -2 | 14 | 4.71* |
| *LC > NC FB* |  |  |  |  |  |  |
|  | Medial prefrontal cortex | -6 | 65 | 25 | 21 | 3.42* |
|  | Inferior frontal gyrus | 30 | 41 | 4 | 74 | 3.99* |
| **Ket+LSD > LSD** | |  |  |  |  |  |
| *HC > NC*  *R2* |  |  |  |  |  |  |
|  | Medial orbitofrontal cortex | 3 | 47 | 4 | 123 | 4.67* |
|  | Lateral orbitofrontal cortex | 12 | 41 | -2 | 15 | 3.82* |

Statistical threshold: p < 0.005 (uncorrected), k > 10, *p < 0.05, FWE after SVC. Montreal Neruological Institute coordinates of peak voxels are given for each cluster. FWE; family-wise error; HC, high conflict; LC, low conflict; NC, no conflict; FB; Feedback processing; R2, final judgment; Pla, placebo; Ket, Ketanserin; SVC, small-volume correction.

**Table S3. Picture ratings in each set in the pre-study survey**

|  | **Set 1** | **Set 2** | **Set 3** |
| --- | --- | --- | --- |
| **Item** | ***M (SD)*** | ***M (SD)*** | ***M (SD)*** |
| Emotional arousal | 1.72 (0.29) | 1.62 (0.34) | 1.60 (0.32) |
| Aesthetic quality | 2.10 (0.40) | 1.97 (0.46) | 2.10 (0.43) |
| Deeper meaning | 1.60 (0.24) | 1.51 (0.29) | 1.50 (0.31) |

No significant difference between ratings of the three sets (all p > 0.05). Each set comprised 60 pictures.

References

1. Dittrich, A. The standardized psychometric assessment of altered states of consciousness (ASCs) in humans. *Pharmacopsychiatry* **31 Suppl 2,** 80–84 (1998).

2. Studerus, E., Gamma, A. & Vollenweider, F. X. Psychometric evaluation of the altered states of consciousness rating scale (OAV). *PloS one* **5,** e12412 (2010).

3. Watson, D., Clark, L. A. & Tellegen, A. Development and validation of brief measures of positive and negative affect: The PANAS scales. *Journal of Personality and Social Psychology* **54,** 1063–1070 (1988).
